# Evaluation of Nutraceutical Properties of Fruits Derived from Some Wild-Growing Plant Species (*Sambucus nigra* L., *Rubia tinctorum* L., *Phytolacca americana* L. and *Sambucus ebulus* L.)

**DOI:** 10.3390/plants15071133

**Published:** 2026-04-07

**Authors:** Constantin Lungoci, Iuliana Motrescu, Laurian Vlase, Ioan Puiu

**Affiliations:** 1 “Ion Ionescu de la Brad” Iasi University of Life Sciences, 3 Sadoveanu Alley, 700490 Iasi, Romania; ioan.puiu@iuls.ro; 2 Iuliu Hațieganu University of Medicine and Pharmacy, 400012 Cluj-Napoca, Romania

**Keywords:** nutraceutics, *Sambucus nigra* L., *Sambucus ebulus* L., *Phytolacca americana* L., *Rubia tinctorum* L.

## Abstract

Wild-growing plant species are sustainable, cost-effective and underexploited sources for bioactive compounds with great nutraceutical potential. In this work, we analysed the fruits of several wild plant species: *Sambucus nigra* L., *Rubia tinctorum* L., *Phytolacca americana* L. and *Sambucus ebulus* L. Liquid chromatography coupled with mass spectrometry revealed diverse concentrations of phenolic acids, with the highest values observed for rutoside (22.183 µg mL^−1^) and isoquercitrin (11.663 µg mL^−1^) in *S. nigra* L., chlorogenic acid (7.422 µg mL^−1^) in *R. tinctorum* L., caftaric acid (4.942 µg mL^−1^) in *P. americana* L., and quercitrin (1.380 µg mL^−1^) and 4-O-caffeoylquinic acid (1.196 µg mL^−1^) in *S. ebulus* L. The spectrophotometric analysis showed that *S. nigra* L. had the highest concentration of total phenols (14.21 mg GAE g^−1^ FW) and the highest flavonoid content (8.07 mg QE g^−1^ FW). The lowest values were recorded for *R. tinctorum* L. (total phenols) and *P. americana* L. (flavonoids). The antioxidant activity was generally high, with the lowest value of 76.08% for *S. nigra* L. and over 90% for all other species, peaking at 93.25% for *P. americana* L. The Trolox Equivalent Antioxidant Capacity (TEAC) assay showed a similar pattern. *S. ebulus* L. and *R. tinctorum* L. exhibited the highest carbohydrate content and protein solubility, respectively. *P*. *americana* L. fruits had the highest ascorbic acid concentration, 0.136 mg g^−1^ FW. These results highlight the remarkable nutraceutical potential of certain wild fruits, identifying them as rich and cost-effective sources of bioactive compounds, particularly antioxidants, with promising perspectives for future studies on their therapeutic potential.

## 1. Introduction

In the current era, marked by rising chronic diseases, the demand for natural bioactive compounds with health-promoting properties has increased. The term “nutraceutical” was first used in scientific language in 1979 by Dr Stephen Defelice, representing the combination of the terms “nutrition” and “pharmaceutical”, highlighting the multiple roles of the compounds included in this category [1]. A more suggestive definition was given by the European Nutraceutical Association, which defines nutraceutical substances as nutritional products with visible, relevant health benefits, not synthetic or chemically formulated for a specific indication, and containing nutrients, at least partially, in concentrated form [2]. The most frequently used classifications of nutraceuticals are based on their origin [2,3], the effect they have in various chronic conditions [4], and chemical nature (e.g., polyphenols, proteins, vitamins, fatty acids, minerals) [5,6].

Dietary patterns rich in nutraceutical compounds derived from plants are increasingly recognised as a key factor in the prevention and management of some diseases, such as cardiovascular disorders, certain forms of cancer, metabolic syndrome and neurodegenerative conditions [6,7,8]. In this context, the concept of nutraceuticals and functional foods has been gaining considerable attention. Fruits are important dietary sources of such bioactive molecules, such as vitamins, phenolic compounds, flavonoids, anthocyanins and other secondary metabolites that can modulate oxidative stress and related pathological processes [4,5,6].

While cultivated fruits such as apples or berries are very common on the nutraceutical market, wild-growing plant species remain largely underexplored. Wild species are a sustainable and cost-effective reservoir of structurally diverse photochemicals. They often experience stronger environmental pressures than domesticated crops and may therefore accumulate higher levels or distinct profiles of phenolic acids, flavonoids and other secondary metabolites [9]. In many regions around the world, fruits from wild plants are traditionally consumed as foods or used as traditional remedies, but their nutraceutical properties are frequently supported only by empirical knowledge, with limited systematic chemical and functional characterisation [9].

Among these, species such as elderberry (*Sambucus nigra* L.), danewort (*Sambucus ebulus* L.), madder (*Rubia tinctorum* L.) and pokeweed (*Phytolacca americana* L.) are of particular interest. They are wild-growing plants with berry-like fruits or drupes that grow in regions such as Romania, often in disturbed areas, roadsides or forests. Despite varying levels of toxicity requiring careful preparation, they are rich in bioactive compounds and have been traditionally used for their benefits.

Elderberry is a part of the *Adoxaceae* family; the genus includes approximately 20 species spread in the subtropical and temperate zones. The most important species is *S. nigra* L, a shrub with a height of 4 to 5 m, having a stem with a very developed pith. The leaves are opposite, imparipinnate (3 to 7 leaflets), with serrate edges pointed at the tip. The flowers are arranged in umbelliform, richly branched cymes. The fruits are spherical, shiny, with three seeds [10]. Elderberry fruits have an analgesic effect and can be used as an adjuvant against migraine, sciatica and neuralgic pain [10,11]. The flowers have diaphoretic, antipyretic and diuretic properties [11]. Furthermore, elderberry has anti-inflammatory and antibacterial properties, being used as a gargle to treat sore throats or as compresses to treat conjunctivitis [12].

Danewort is also a part of the *Adoxaceae* family, being an herbaceous, perennial species that forms vigorous bushes up to 1–2 m tall, with an unpleasant odour. In the soil, it has vigorous, trailing, fast-growing rhizomes. The aerial stem is notched, rigid and green. The leaves are imparipinnate, opposite, with 7 to 11 ovate-lanceolate leaflets. The flowers are arranged in corymbiform cymes. Mature fruits are black, spherical berries, with a persistent calyx [13,14]. Danewort berries are rich in polyphenols, anthocyanins, triterpenes, tannins, iridoid glycosides, cardiac glycosides, caffeic acid derivatives, chlorogenic acid, ursolic acid and lectins [15]. Reports in the literature report its use in alternative medicine to treat haemorrhoids, rheumatic pain, cold, fever, snakebites and wounds. The root is also used in several countries in Asia and Europe to cure various ailments, including kidney stones and bladder diseases [16,17].

Madder is part of the *Rubiaceae* family, a genus including approximately 70 species [18]. Herbs, sprawling to climbing, perennial, with extensive stout, woody, and with red rhizomes, madder plants have long stems, 1 to 2.5 m long, often fascicled, quadrangular with sharp angles, somewhat retrorsely aculeolate or glabrous. Leaves are organised in whorls of 4–6, shortly petiolate to subsessile. The inflorescences are thyrsoid, leaflike with many-flowered cymes, terminal and axillary from upper stem nodes. The peduncles are up to 50 mm, with narrowly elliptic bracts of 2 to 5 mm [19].

Madder has anti-inflammatory, antioxidant, hepatoprotective and antibacterial properties, confirmed by both in vivo and in vitro studies [20]. A survey conducted in Morocco on the use of these plants in ethnomedicine revealed that this species is frequently used in the treatment of hypertension and other heart conditions [21]. The roots are frequently used as dyes due to their high anthraquinone content [22].

Pokeweed is a member of the *Phytolaccaceae* family, a genus of about 70 species distributed worldwide from Western and Northern Europe to temperate Asia, Africa, the Himalayas and some regions from Mexico and Tropical America [23]. The plant grows 1 to 4 m tall, with a large cabbage-like root. Its leaves are alternate, ovate-elliptic, pointed, smooth-edged and 10 to 40 cm long, frequently reddish in colour, with a stout midrib and a short petiole. Flowers are relatively small and borne in narrow racemes 10 to 15 cm long that cluster along the stem; there is no corolla, and each flower has 4 to 5 sepals that can be white, reddish, or greenish, 8 to 10 stamens, and 8 to 10 styles. The fruit is a depressed-globular fleshy berry with a diameter of about 1 cm, dark red to nearly black at maturity, and it is formed from 10 carpels and filled with deep red juice. Each carpel contains a single lens-shaped seed. The berries are mildly sweet and essentially odourless [24].

Wild fruits are often overlooked in modern agriculture; despite their abundance in regions like Eastern Europe, including Romania’s biodiverse Carpathian foothills and Danube Delta, their nutraceutical potential has been scarcely studied. Some studies exist about *Vaccinium myrtillus* and *Vaccinium corymbosum* [25,26], *Rosa canina* L., *Prunus spinosa* L., *Vaccinium vitis-idaea* L., *Cornus mas* L. [9,27], *Rubus fruticosus* L. and *Rubus laciniatus* L. [28], *Ribes grossularia* L., *Prunus cerasus* L., *Fragaria x ananassa* Duch., *Hippophae rhamnoides* L. [29], *Fragaria vesca* L., *Rubus idaeus* L., *Rubus fruticosus* L. [30] and other species. A comprehensive evaluation of the nutraceutical potential of the fruits from such wild-growing plants is therefore timely for several reasons. Firstly, it can provide scientific support for their inclusion as alternative or complementary sources of bioactive ingredients in functional foods, nutraceutical formulations or phytopharmaceuticals. Secondly, it contributes to the valorisation and conservation of local biodiversity, highlighting the added value of wild species. Thirdly, it offers a rational basis for reassessing traditional uses and for guiding future in vivo and clinical studies on their therapeutic potential.

In this context, this study aims to evaluate the nutraceutical potential of fruits derived from these locally abundant sources in the spontaneous flora and provide an overview of the most important nutraceutical parameters. The studied wild fruit species were characterised in terms of selected phenolic acids and flavonoids by liquid chromatography coupled with mass spectrometry, quantified total phenolic and flavonoid contents and assessed antioxidant capacity using DPPH and TEAC assays. In addition, basic nutritional parameters such as carbohydrate content, soluble proteins and ascorbic acid were determined. By providing a comparative overview of these chemical and functional traits, this work seeks to highlight the potential of these wild fruits as new, rich and cost-effective bioactive sources that can be isolated and integrated into a series of new nutraceutical products, with benefits for some local communities in harnessing biodiversity for health and sustainability.

## 2. Results

### 2.1. LC-MS/MS Analysis

**Figure 1:**
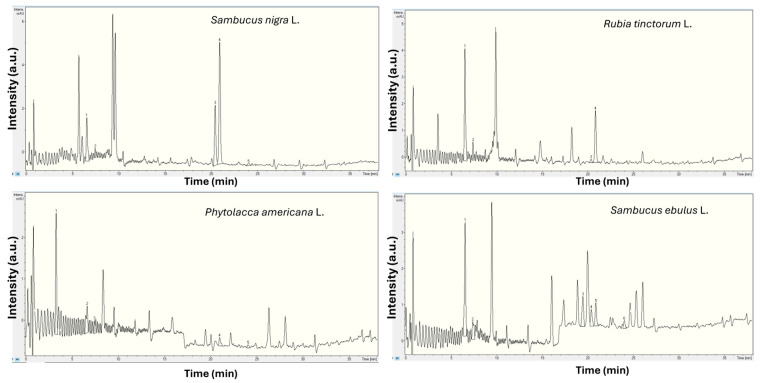
Comparative UV chromatogram of polyphenols for the 4 analysed species.

From the liquid chromatography analysis presented in Figure 1 and Table 1, several phenolic acids were identified and quantified, with different concentrations in all analysed samples. Thus, for danewort, there was a large diversity of compounds, while the highest detected concentrations were found for elderberry. For madder, only four of the seven acids were found, while in pokeweed, there were only five.

Caftaric acid was detected only in pokeweed with a concentration of 4.942 μg mL^−1^ (Table 1). Chlorogenic acid and 4-O-caffeoylquinic acid were present for all species with variable concentrations ranging between 0.240 and 7.422 μg mL^−1^. The flavonoid hyperoside was identified only in the species danewort, while isoquercitrin was identified in all species except pokeweed.

### 2.2. Total Phenolic Content

**Figure 2:**
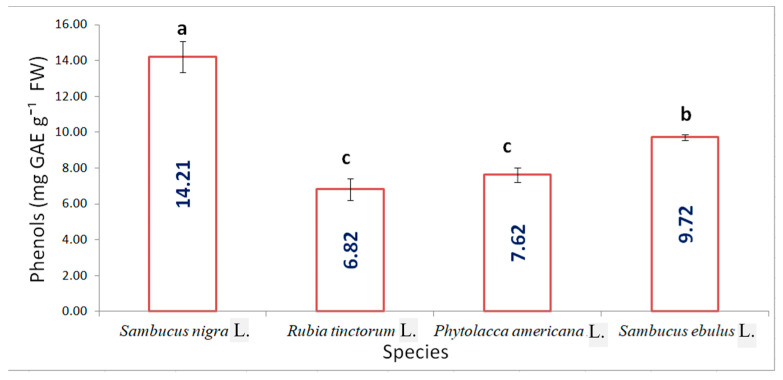
Total polyphenols in the fruits from the studied species (results are averages with standard deviations indicated on the chart, letters showing significant differences at the 0.05 level).

The total polyphenol content analysed by the spectrophotometric method, shown in Figure 2, indicated a high variability between species. Similar values were recorded for madder (6.82 mg GA g^−1^ FW) and pokeweed (7.62 mg GA g^−1^ FW), with insignificant differences. Despite belonging to the same family, there were significant differences between the total polyphenols in *Sambucus* sp. For elderberry, the concentration was the highest, 14.21 mg GA g^−1^ FW.

### 2.3. Total Flavonoid Content

**Figure 3:**
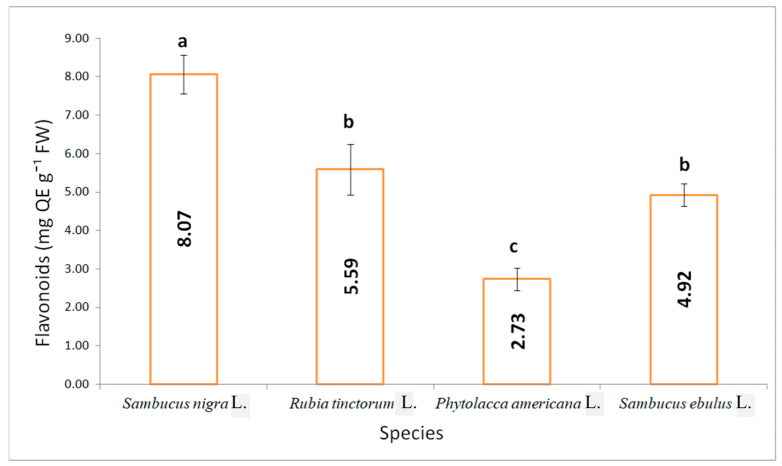
Flavonoid content in the fruits of the studied species (results are averages with standard deviations indicated on the chart, letters showing significant differences at the 0.05 level).

Flavonoids, a subgroup of phenolic compounds with a protective role, were recorded in various concentrations for the studied species, as it is presented in Figure 3. There were no significant differences between madder and danewort variants. The lowest concentration of flavonoids was determined for pokeweed, of 2.72 mg QE g^−1^ FW, while the highest was for elderberry, of 8.07 mg QE g^−1^ FW.

### 2.4. Antioxidant Activity with DPPH Assay

**Figure 4:**
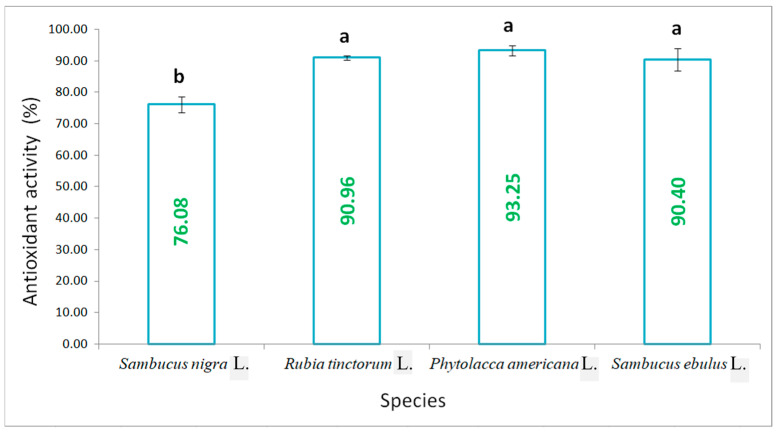
Antioxidant activity of fruit extracts corresponding with the four studied species determined with the DPPH assay (results are averages with standard deviations indicated on the chart, letters showing significant differences at the 0.05 level).

Total neutralisation of reactive oxygen species (ROS) using the DPPH reagent highlighted that, for the species madder, pokeweed and danewort, the neutralisation degree had the highest percentage, the values ranging between 90.96% and 93.25%, with no significant differences in between. A lower antioxidant activity was determined for elderberry, of only 76.08%, as it is presented in Figure 4.

### 2.5. Trolox Equivalent Antioxidant Capacity (TEAC) Assay

**Figure 5:**
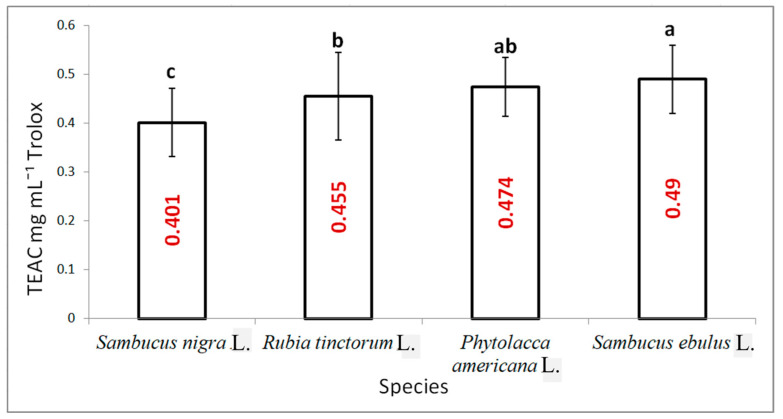
Antioxidant activity of fruit extracts corresponding with the four studied species determined with the TEAC assay (results are averages with standard deviations indicated on the chart, letters showing significant differences at the 0.05 level).

The second assay used to quantify the antioxidant activity based on the neutralisation of the ABTS radical generated with the help of potassium persulfate indicated close values with significant statistical differences between the analysed samples (Figure 5). The highest value of antioxidant activity was determined for danewort with a value of 0.49 mg mL^−1^ Trolox. The smallest value was determined for elderberry.

### 2.6. Total Soluble Sugar

**Figure 6:**
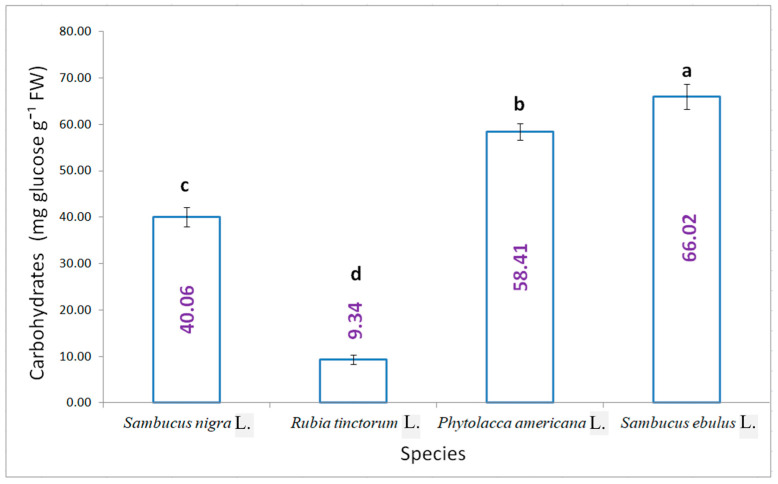
The content of soluble sugar in the studied samples (results are averages with standard deviations indicated on the chart, letters showing significant differences at the 0.05 level).

The soluble carbohydrate content presented in Figure 6 shows significant differences between the four analysed variants. The highest content was determined for danewort, 66.02 mg glucose g^−1^ FW, followed by pokeweed, 58.41 mg glucose g^−1^ FW. Lower values were obtained for the other two species.

### 2.7. Bradford Protein Assay Results

**Figure 7:**
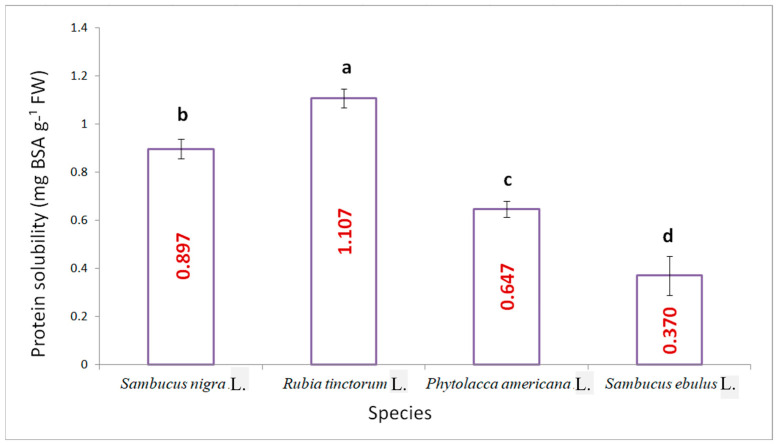
The content of soluble proteins for the studied variants (results are averages with standard deviations indicated on the chart, letters showing significant differences at the 0.05 level).

The highest amount of soluble protein (Figure 7) was recorded for the madder variant with a value of 1.107 mg BSA g^−1^ FW, followed by the elderberry variant with 0.987 mg BSA g^−1^ FW, and the other two species with statistically significant differences, as low as 0.370 mg BSA g^−1^ FW for danewort.

### 2.8. Ascorbic Acid Content

**Figure 8:**
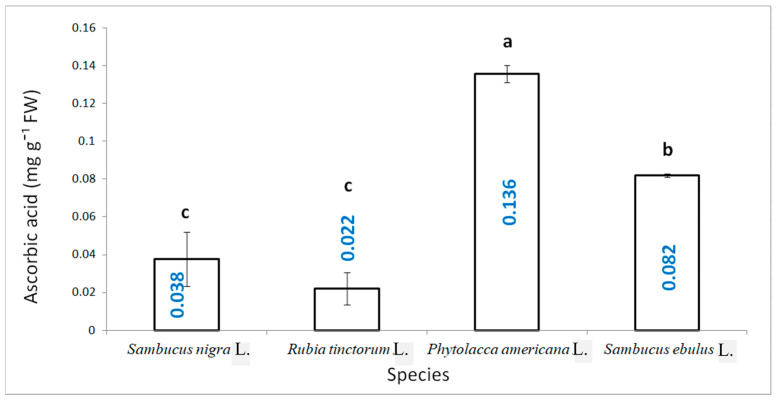
The ascorbic acid content for the studied species (results are averages with standard deviations indicated on the chart, letters showing significant differences at the 0.05 level).

The ascorbic acid content was determined using the spectrophotometric method, with a standard curve made for high-purity ascorbic acid. The highest amount of vitamin C was determined in pokeweed fruits with a value of 0.136 AC mg g^−1^ FW, followed by danewort, 0.082 AC mg g^−1^ FW, and significantly smaller values for the other two species, the lowest concentrations being recorded for madder of 0.022 AC mg g^−1^ FW, with no statistically significant differences between the latter species (Figure 8).

### 2.9. Pearson Correlation

Pearson correlation coefficients were determined for the studied parameters (Table 2). The correlation table presents some strong positive correlations between phenols, flavonoids, ascorbic acid and antioxidant activity, as well as between the two methods used to evaluate the latter [31]. Out of the total correlation coefficients, thirteen values were not statistically significant, while ten showed significant and highly significant negative correlations.

## 3. Discussion

Secondary metabolites have multiple roles in plants, ensuring the protection against stress factors, are useful for attracting pollinators, rhizosphere-specific microflora and vectors for seed dispersal [32]. Among these compounds, polyphenols, a category that includes phenolic acids and flavonoids, play a significant role, representing approximately 40% of the total organic carbon circulating in the biosphere [33]. The primary compounds resulting from the photosynthetic process follow the phenyl-propanoid pathway of the shikimic cycle to synthesise these secondary metabolites, including those highlighted in our study [34]. Phenols are polar compounds; therefore, their extraction was performed in 70% alcohol [35]. Caftaric acid is a phenolic compound derived from the hydroxycinnamic acid cycle [36], frequently found in juicy and colourful fruits, especially grapes [37]. The literature mentions it having hypoglycaemic and hypotensive properties [38], as well as numerous pharmacological effects such as anti-inflammatory, anti-oxidant, antimutagenic, hepatoprotective, anticancer and neuroprotective [38,39]. The value recorded for pokeweed fruits seems to correspond with an increased antioxidant activity level, as found in this study. The value is high compared to other extracts; however, lower compared to other studies investigating extracts of *Cichorium intibus* L. and roots of *Echinacea purpurea* L. [40].

From our analyses, we found that chlorogenic acid was present in all studied species. It is a polyphenolic phenylacrylate compound quite common in the plant kingdom. The most important known biological activities of chlorogenic acid are kidney and liver protection, antioxidant, antibacterial, anti-inflammatory, antiviral, and antitumor effects, and regulation of sugar and lipid metabolism [41,42]. There are numerous studies regarding the presence of this acid in the studied plants [43,44,45,46]. One study found similar values for the chlorogenic acid content, analysing the nutraceutical profile of the juice obtained from elderberry fruits [43].

4-O-caffeoylquinic acid is frequently found in coffee beans, in a percentage of about 5% [47]. In our study, the highest content was found in the fruits of danewort, 1.196 μg/mL. Due to the large number of hydroxyl groups, this compound’s antioxidant potential comes from donating electrons or hydrogen atoms to free radicals and reducing their reactivity; it has been shown that it can protect against oxidative stress and inflammation, therefore being an important nutraceutical compound [48]. A study conducted on pokeweed highlighted that the use of water as a solvent for extraction leads to higher concentrations (110.05 μg g^−1^) of this acid than the alcoholic extraction (72.13 μg g^−1^) [49].

Hyperoside is a flavonoid found in many plant families, such as *Rosaceae*, *Hypericaceae* and *Fabaceae* [50]. It has a wide spectrum of biological activities, including anti-inflammatory, anticancer, antiviral, antibacterial, antidepressant and organo-protective. These pharmacological properties underline its use in the treatment of several diseases such as sepsis, arthritis, colitis, diabetic nephropathy, myocardial ischemia-reperfusion, pulmonary fibrosis and cancers [51]. There is a lack of studies of this compound, and among the species we studied, results are found only for danewort.

Isoquercitrin (quercitrin-3-O-β-d-glucopyranoside) is a flavonoid found in many plants; for the production of drugs and nutraceuticals, its use is based on enzymatic hydrolysis with precursors. The literature mentions its presence naturally in species such as *Hypericum perforatum* L., *Cercis candensis* L., *Eucommia ulmoides* Oliv., *Caragana arborescens* Lam. and *Allium fistulosum* L. [52,53]. It has demonstrated its efficacy both in vitro and in vivo, with actions against oxidative stress, cardiovascular disorders, cancer, allergic reactions and diabetes. It has a stable passage through the liver, being found intact in plasma and tissues [54]. The highest content of isoquercitrin was identified in *Sambucus nigra* L. fruits. The diversity of polyphenolic compounds in this species and their prebiotic role was also highlighted by other authors [55].

Rutoside, also known as vitamin P, is a flavonoid frequently used as a nutraceutical substance. Currently, over 860 products containing this compound exist [56]. In our study, the highest content was identified in elderberry, 22.183 μg mL^−1^, and madder, 7.709 μg mL^−1^. Large contents were also found in species such as *Fagopyrum esculentum* Monech. (*Polygonaceae*), *Ruta graveoles* L. (*Rutaceae*), *Sophora japonica* L. (*Fabaceae*) and *Eucalyptus* spp. (*Myrtaceae*). The main disadvantage associated with rutin is its poor bioavailability, mainly caused by its low solubility in water, poor stability and limited membrane permeability [57]. Conventionally, rutin is used as an antifungal, antimicrobial and antiallergic agent. Current research has demonstrated its pharmacological benefits for the treatment of various chronic diseases such as diabetes, cancer, hypercholesterolemia and hypertension [58].

Quercitrin is a glycosylated flavanol frequently found in the plant world in species such as *Melastoma malabathricum* L., in which the reported content was 36.02 mg g^−1^ [59]. A study conducted in 2019 highlighted the fact that quercitrin extracted from *Copaifera langsdorffii* Desf. has a preventive effect on the formation of kidney stones [60], while the one extracted from *Fagopyrum tataricum* (L.) Gaertn. proved to promote apoptosis, inhibited proliferation of several cancer cell lines, and strongly inhibited infection, inflammation, and oxidative stress [61].

The analysis of phenols and flavonoids had as a main purpose the use of the extracts in the nutraceutical field. The antioxidant and anti-inflammatory effects of these compounds, and the proven qualities in the prevention of chronic diseases, are well known [62]. The peculiarity of our study is that the analysis of the phenolic compounds was carried out directly on fruits and not a made product. For example, a study highlighting the nutraceutical role of the juice of elderberry fruits reported a content of the phenolic compounds in the juice much higher compared to our results of alcoholic fruit extracts [43].

The antioxidant properties of fruit extracts analysed in this study were determined by two methods, providing a greater precision in establishing the antioxidant effect of the analysed secondary metabolites. The fruits of the analysed species exhibit notable antioxidant activity reported in the literature, primarily driven by polyphenols, anthocyanins and flavonoids, though data is most robust for elderberries and danewort [55,63]. Elderberry studies have found strong free radical scavenging, with DPPH values between 82.08 and 89.25% in fruit extracts, slightly higher than the value we found [12]. Danewort fruits exhibited an antioxidant activity up to about IC_50_ 190 μg mL^−1^, with a value of the total flavonoid content comparable with the one we found 8.62 mg QE g^−1^ extract [56]. It was attributed to phenols, flavonoids and anthocyanins, with the highest values of the antioxidant activity of fruits compared to other parts of the plant. For madder, there are a very limited number of reports; aerial and root extracts were shown to display solid DPPH inhibition (66 to 83%) and FRAP/TAC activity [45]. Pokeweed berries demonstrated high DPPH radical scavenging, outperforming some standards in some in vivo assays, mainly due to the presence of phenolic acids [64].

Ascorbic acid is one of the most important antioxidants used in the prevention and treatment of chronic diseases. Its physiological role is much broader, encompassing different processes, from facilitating iron absorption, involvement in hormone and carnitine synthesis, to important roles in epigenetic processes [65]. The effects of vitamin C administration on cancer, cardiovascular diseases and infections are quite minor or even questionable in the general population, which is why it is mostly used for prevention rather than for treatment. In high doses, it acts more as a prooxidant than as an antioxidant [66]. The literature reports regarding the vitamin C content in the studied fruits are limited and variable. Elderberry and danewort seem to exhibit higher concentrations of vitamin C than the other species. For the first, amounts of 6 to 25 mg 100 g^−1^ FW were found in the fresh fruits [67], while other studies found concentrations up to 44.26 mg 100 g^−1^ FW depending on the cultivar [68]. For danewort and madder, we could not find reliable quantitative data. Pokeweed berries contain vitamin C, but the species is not so much studied, so the amount is unquantified in the existing literature, to the best of our knowledge. Based on our results, we must underline the importance and necessity of a detailed analysis of these species for their nutraceutical power. At the same time, looking at the Pearson correlation matrix, there is a positive correlation of the antioxidant activity (both methods) with ascorbic acid rather than with other monitored antioxidant compounds (Table 2).

The analysis of soluble protein and carbohydrate content provides information regarding their availability as nutritional resources. Furthermore, soluble proteins serve as a standard reference for enzyme evaluation. They also play a direct role in the antioxidant activity of plants through enzymes such as catalase, superoxide dismutase (SOD) and peroxidase, which neutralise reactive oxygen species [69]. These analyses assist in generating data on soluble proteins and digestible carbohydrates necessary for studying nutritional ecology, plant interactions and food web dynamics, which, in turn, will enhance physiological and ecological research [69,70].

The results of this study align with the existing literature regarding the great bioactive potential of some wild species and underutilised plants. The high concentrations of phenols, flavonoids and antioxidants in general identified in fruits from elderberry, pokeweed, madder and danewort are comparable with the profiles reported for other similar plants. The analysed fruits show increased antioxidant and vitamin levels, highlighting their potential for medical and food industry applications. These high secondary metabolite concentrations correlate with enhanced ecological adaptability and stress resilience and prove them as valuable sources for the development of nutraceutical products and functional foods, underlining the importance of using this less explored potential of wild species.

## 4. Materials and Methods

### 4.1. Plant Material and Extraction Procedure

Fruits from the four studied species were harvested at full maturity from two nearby locations: Anastasie Fatu Botanical Garden in Iasi (lat. 47°18′62.85″ N, long. 27°55′55.61″ E) for danewort and elderberry, and the Medicinal, Aromatic, and Spice Plant Collection of USV Iasi (lat. 47°18′95.95″ N, long. 27°55′ 55.61″ E) for pokeweed and madder. The samples were cold-preserved to prevent enzymatic degradation and the loss of vitamin C. For analysis, the pulp was separated from the seeds, and 0.5 g of each sample was incubated with 10 mL ethyl alcohol for 24 h [71].

### 4.2. LC-MS/MS Protocol

The phytochemical profiles of the ethanol extracts were analysed using an Agilent 1100 HPLC Series system (Agilent, Santa Clara, CA, USA) consisting of a binary gradient pump, autosampler, degasser and column thermostat. The system was coupled with a UV detector and an Agilent Ion Trap 1100 SL mass spectrometer (LC/MSD Ion Trap VL) (Agilent Technologies, Santa Clara, CA, USA) equipped with electrospray ionisation (ESI) and atmospheric pressure chemical ionisation (APCI) sources. Chromatographic separation was achieved on a Zorbax SB-C18 column (100 × 3.0 mm i.d., 3.5 μm particle size, Agilent), maintained at 48 °C. The injection volume was 5 µL at a constant flow rate of 1 mL/min. The analytical protocol was based on previously validated methods [72,73,74].

The analytical method was employed for the analysis of 23 polyphenolic compounds [73,75]. The mobile phase was a mixture of methanol and 0.1% acetic acid (*v*/*v*), with a linear gradient from 5% to 42% methanol over 35 min, followed by a 3-min isocratic elution at 42%. Phenolic acids were monitored at 330 nm (0–17 min), and flavonoids/aglycones at 370 nm (17–38 min) [72,74,75]. Compound identification was performed based on the specific MS or MS/MS fragmentation patterns and mass spectra, while quantitative analysis was performed based on the UV spectra after positive qualitative identification based on MS data. Detailed information regarding the retention time (min), analysis mode (ion source, polarity, transition type), molecular ion and dauther ions are presented in the Supplementary Material Table S1.

All analyses were performed in triplicate, and results are expressed as mean values ± standard deviation (SD). Identification was confirmed by retention time and mass spectra comparison against a library of analytical standards. Quantification was carried out via the external standard method; five-point calibration curves (0.5–50 µg mL^−1^) showed excellent linearity (R^2^ > 0.999). Detection limits (LOD) were defined as the concentration yielding a signal-to-noise ratio > 3. Data collection and processing were performed using DataAnalysis and ChemStation software (Agilent, Santa Clara, CA, USA).

### 4.3. Total Phenolic Content

Total polyphenol content (TPC) was measured by the Folin–Ciocalteu assay. Briefly, 100 μL of alcoholic extract was combined with 100 μL Folin–Ciocalteu reagent in an Eppendorf tube. After 2 min, 800 μL of 5% (*w*/*v*) Na_2_CO_3_ was added and the mixture was incubated at 40 °C for 20 min. Absorbance was then read at 740 nm on a UV-Vis SP-UV 1100 spectrophotometer (DLAB Scientific Co., Ltd., Beijing, China). TPC was determined from a gallic acid (GA) calibration curve and reported as mg gallic acid equivalents (GAE) per L (r^2^ = 0.9947) [71].

### 4.4. Total Flavonoid Content

Flavonoid content was measured by mixing 0.25 mL of plant extract with 5% NaNO_2_ and 10% AlCl_3_, adjusting to basic pH with 1 M NaOH. Absorbance was read at 510 nm, and flavonoid levels were calculated from a quercetin calibration curve (r^2^ = 0.9912) [76].

### 4.5. Evaluation of the Antioxidant Activity Using the DPPH Assay

The antioxidant activity was assessed by the DPPH assay. A 2,2-diphenyl-1-picrylhydrazyl (DPPH) (Sigma-Aldrich, Bucharest，Romania) working solution was prepared by dissolving 24 mg DPPH in 550 mL methanol and allowing it to react in the dark at room temperature for 24 h until an absorbance of 1.1 ± 0.2 at 515 nm was reached. Extracts were diluted 1:25 in water, and 150 µL of the diluted extract was mixed with 2850 µL of the DPPH solution. After 30 min incubation, absorbance at 515 nm was measured using a DLAB UV-Vis SP-UV 1100 spectrophotometer (DLAB Scientific Co., Ltd., Beijing, China). The percent inhibition of the DPPH radical was then calculated according to the equation:

$$\%\;DPPH\;radical\;scaverging\;activity\;=\;\frac{ABSc\;-\;ABSs}{ABSs}\;\times \;100$$

where ABSc is the absorbance of the blank solution at 515 nm, and ABSs is the absorbance of the sample after 30 min of incubation with DPPH at 515 nm [77].

### 4.6. Evaluation of the Antioxidant Activity Using the TEAC Assay

ABTS•+ radicals were produced following the original method by mixing equal volumes of substrate solution (2,2′-azino-bis(3-ethylbenzothiazoline-6-sulfonic acid), 7 mM) and oxidant (potassium persulfate, 2.45 mM) and allowing the reaction to proceed in the dark for 12 h. The resulting radical solution was diluted with ethanol or with pH 3.6 and pH 7.4 buffers to an absorbance of 0.70 ± 0.10 at 734 nm. Aliquots of test compound (40 µL) were placed in test tubes, then 4 mL of the radical solution was added. Reactions were incubated for a standard time of 6 min, and in some experiments for an extended time of 30 min, after which absorbance was measured again at 734 nm. All assays were performed in 3 replications [78].

### 4.7. Determination of the Total Soluble Sugars

Total soluble sugars were measured following a previously described method with minor modifications. Approximately 0.1 g of ground plant tissue was homogenised in 10 mL ice-cold 80% acetone. The mixture was centrifuged at 10,000 rpm for 10 min at 4 °C, and 1 mL of the supernatant was mixed with 3 mL anthrone reagent (0.15% in 96% H_2_SO_4_). Samples were heated in a 95 °C boiling water bath for 15 min, then cooled on ice. Absorbance was measured at 620 nm using a visible spectrophotometer (DLAB UV-Vis SP-UV 1100DLAB Scientific Co., Ltd., Beijing, China). Total soluble sugar content was calculated from a glucose standard curve and expressed as mg L^−1^ (r^2^ = 0.9909) [79].

### 4.8. Bradford Protein Assay

Protein concentration was determined by the Bradford assay. To each test tube, 0.1 mL of fruit extract obtained through grinding in potassium phosphate buffer (pH 7.8) was mixed with 5 mL of Bradford reagent (Supelco), shaken, and the absorbance was read at 595 nm after 2 min. Bovine serum albumin (BSA) (Sigma-Aldrich) served as the standard, and protein amounts are reported as mg BSA equivalent g^−1^ fresh weight [80].

### 4.9. Determination of L-Ascorbic Acid Content

About 0.1 g of pulp was weighed into a mortar, and 2 mL of 10% (*v*/*v*) trichloroacetic acid was added. The sample was homogenised with a pestle under dim light and on ice. The homogenate was centrifuged at 5000 rpm for 10 min at 4 °C. Then, 0.3 mL of the supernatant was mixed with 0.2 mL of 10% (*v*/*v*) Folin–Ciocalteu reagent and 1.7 mL of distilled water. Absorbance was read at 760 nm using a UV–Vis spectrophotometer (DLAB Scientific Co., Ltd., Beijing, China). A calibration curve was prepared with ascorbic acid standards (0–6 mg L^−1^; r^2^ = 0.9903) [81]. Measurements were performed in triplicate.

### 4.10. Statistical Processing

Pearson correlation coefficients were calculated in Microsoft Excel 2022 to assess relationships among measured and derived traits. One-way analysis of variance (ANOVA) was performed using SPSS version 20.0 (IBM Corp., Chicago, IL, USA). Results are reported as mean ± standard deviation (SD), and differences were considered statistically significant at *p* < 0.05 [31,82].

## 5. Conclusions

Spontaneous flora represents an important biological source, with considerable valorisation potential, due to its phytochemical diversity and high concentrations of secondary metabolites with nutraceutical relevance. However, this resource is still insufficiently explored and underutilised for possible applications in food, pharmaceutical and natural supplement industries.

The results of the present study indicate that the fruits of the four studied species could serve as important sources of raw materials for the extraction of bioactive compounds, considering both their low acquisition costs, natural availability and high nutrient and phytochemical values. Among the studied species, elderberry stands out as an especially valuable nutraceutical resource, combining the highest levels of phenolic compounds and flavonoids, with a strong antioxidant capacity. Pokeweed showed exceptional antioxidant activity and ascorbic acid content, while danewort and madder provide notable carbohydrate and protein fractions. Together, they offer complementary profiles of phenolic acids and basic nutrients, which recommend them as good candidates for developing functional ingredients. It is well-established that, at least, our country has areas with low pollution levels and high species diversity, factors that ensure the quality of the raw materials with enhanced potential for subsequent use in applications. The results obtained in this study provide a partial evaluation of some bioactive compounds that can be harnessed from these species and may be useful for the development of innovative natural products. From this perspective, our study supports the need for future research focusing on optimising extraction technologies and reducing production costs, so that the use of these plant resources becomes not only scientifically justified, but also economically viable and sustainable in the long term.

## Figures and Tables

**Table 1:** Phenolic profile of ethanol extracts evaluated by LC-MS/MS (concentration in sample µg/mL).

Compounds (µg mL^−1^)	Species			
	*Sambucus nigra* L.	*Rubia tinctorum* L.	*Phytolacca americana* L.	*Sambucus ebulus* L.
*Caftaric acid*	nd	nd	4.942 ± 0.098	nd
*Chlorogenic acid*	4.025 ± 0.241	7.422 ± 0.148	1.760 ± 0.088	5.912 ± 0.236
*4-O-caffeoylquinic acid*	0.431 ± 0.017	0.622 ± 0.018	0.240 ± 0.016	1.196 ± 0.011
*Hyperoside*	nd	nd	nd	2.564 ± 0.153
*Isoquercitrin*	11.663 ± 0.466	0.876 ± 0.014	nd	2.417 ± 0.024
*Rutoside*	22.183 ± 0.443	7.709 ± 0.231	0.658 ± 0.016	2.514 ± 0.125
*Quercitrin*	0.912 ± 0.009	nd	0.445 ± 0.041	1.380 ± 0.134

nd—not detected.

**Table 2:** Pearson correlation matrix between the studied factors (* correlation is significant at the 0.05 level; ** correlation is significant at the 0.01 level (2-tailed)). Strong positive correlations are indicated in red colour, while strong negative corelations in blue.

	Species	Phenols	Flavonoids	DPPH	TEAC	Soluble Sugars	Protein	Ascorbic Acid
Species	1							
Phenols	0.080	1						
Flavonoids	0.675 *	0.754 **	1					
DPPH	−0.336	−0.885 **	−0.858 **	1				
TEAC	−0.481	−0.731 **	−0.805 **	0.852 **	1			
Soluble sugars	−0.884 **	0.200	−0.400	0.121	0.422	1		
Protein	0.765 **	−0.037	−0.433	−0.255	−0.625 *	−0.937 **	1	
Ascorbic acid	−0.963 **	−0.282	−0.786 **	0.477	0.572	0.761 **	−0.669 **	1

## Data Availability

Data is available upon request from the authors.

## Supplementary Materials

The following supporting information can be downloaded at: https://www.mdpi.com/article/10.3390/plants15071133/s1, Table S1: Chromatographic retention times and MS analysis mode of monitored polyphenols.

## Abbreviations

The following abbreviations are used in this manuscript:

TEAC	Trolox Equivalent Antioxidant Capacity
GAE	Gallic acid equivalents
QE	Quercitrin equivalents
LC-MS/MS	Liquid chromatography-mass spectrometry/mass spectrometry
FW	Fresh weight
DPPH	2,2-diphenyl-1-picrylhydrazyl
ROS	Reactive oxygen species
ABTS	2,2′-azino-bis(3-ethylbenzothiazoline-6-sulfonic acid)
HPLC	High performance liquid chromatography
BSA	Bovine serum albumin
SOD	superoxide dismutase
