# Supplementary material for: Evaluation of Nutraceutical Properties of Fruits Derived from Some Wild-Growing Plant Species (Sambucus nigra L., Rubia tinctorum L., Phytolacca americana L. and Sambucus ebulus L.)
Source: Plants (Basel). 2026 Apr 7;15(7):1133. doi: 10.3390/plants15071133 (PMC13074962; doi:10.3390/plants15071133)
Supplement: Supplementary file 1 [file plants-15-01133-s001.zip › plants-4210729-supplementary.pdf]

Supplementary Material

Table S1. Chromatographic retention times and MS analysis mode of monitored polyphenols

| No. | Compound name | Molecular formula | Retention time (min) | Analysis mode (ion source, polarity, transition type) | Molecular ion | Dauther ions |
|---|---|---|---|---|---|---|
| 1 | Caftaric acid | $C_{13}H_{12}O_9$ | 2.15 | ESI-, MRM | 311 | 179 |
| 2 | Gentisic acid | $C_7H_6O_4$ | 2.72 | ESI-, MRM | 153 | 109 |
| 3 | Caffeic acid | $C_9H_8O_4$ | 5.85 | ESI-, MRM | 179 | 135 |
| 4 | Chlorogenic acid | $C_{16}H_{18}O_9$ | 6.63 | ESI-, MRM | 353 | 191 |
| 5 | 4-*O*-Caffeoylquinic acid | $C_{16}H_{18}O_9$ | 7 | ESI-, MRM | 353 | 173 |
| 6 | *p*-Coumaric acid | $C_9H_8O_3$ | 9.15 | ESI-, MRM | 163 | 119 |
| 7 | Ferulic acid | $C_{10}H_{10}O_4$ | 12.43 | ESI-, MRM | 193 | 134 |
| 8 | Sinapic acid | $C_{11}H_{12}O_5$ | 14.67 | ESI-, MRM | 223 | 149 |
| 9 | Vitexin | $C_{21}H_{20}O_{10}$ | 18 | ESI-, MRM | 431 | 311 |
| 10 | Hyperoside | $C_{21}H_{20}O_{12}$ | 18.96 | ESI-, MRM | 463 | 301 |
| 11 | Vitexin 2-O-rhamnoside | $C_{27}H_{30}O_{14}$ | 19.4 | ESI-, MRM | 578 | 431 |
| 12 | Isoquercitrin | $C_{21}H_{20}O_{12}$ | 19.9 | ESI-, MRM | 463 | 301 |
| 13 | Rutin | $C_{27}H_{30}O_{16}$ | 20.4 | ESI-, MRM | 609 | 301 |
| 14 | Myricetin | $C_{15}H_{10}O_8$ | 21.1 | ESI-, SIM | 317 | 317 |
| 15 | Fisetin | $C_{15}H_{10}O_6$ | 22.8 | ESI-, SIM | 285 | 285 |
| 16 | Quercitrin | $C_{21}H_{20}O_{11}$ | 23.26 | ESI-, MRM | 447 | 301 |
| 17 | Kaempferitrin | $C_{27}H_{30}O_{14}$ | 25.6 | ESI-, MRM | 577 | 285 |
| 18 | Quercetin | $C_{15}H_{10}O_7$ | 26.82 | ESI-, SIM | 301 | 301 |
| 19 | Kaempferol 3-rhamnoside | $C_{21}H_{20}O_{10}$ | 27.4 | ESI-, MRM | 431 | 285 |
| 20 | Patuletin | $C_{16}H_{12}O_8$ | 28.74 | ESI-, SIM | 331 | 331 |
| 21 | Luteolin | $C_{15}H_{10}O_6$ | 29.24 | ESI-, SIM | 285 | 285 |
| 22 | Kaempferol | $C_{15}H_{10}O_6$ | 31.73 | ESI-, SIM | 285 | 285 |
| 23 | Apigenin | $C_{15}H_{10}O_5$ | 33.24 | ESI-, SIM | 269 | 269 |
